# Etodolac and the risk of acute pancreatitis

**DOI:** 10.1051/bmdcn/2017070104

**Published:** 2017-03-03

**Authors:** Kuan-Fu Liao, Kao-Chi Cheng, Cheng-Li Lin, Shih-Wei Lai

**Affiliations:** 1 College of Medicine, Tzu Chi University Hualien 970 Taiwan; 2 Department of Internal Medicine, Taichung Tzu Chi General Hospital Taichung 427 Taiwan; 3 Graduate Institute of Integrated Medicine, China Medical University Taichung 404 Taiwan; 4 College of Medicine, China Medical University Taichung 404 Taiwan; 5 Department of Family Medicine, China Medical University Hospital Taichung 404 Taiwan; 6 Management Office for Health Data, China Medical University Hospital Taichung 404 Taiwan

**Keywords:** Acute pancreatitis, Etodolac

## Abstract

Objective: The aim of this study was to explore the association between etodolac use and acute in Taiwan.

Design: We designed a case-control study using the database of Taiwan’s National Health Insurance.

Subjects: In all, 7577 subjects aged 20 years or older with newly diagnosed acute pancreatitis were defined as cases, and 27032 sex-matched and age-matched subjects without acute pancreatitis were defined as controls. The period considered for this study was from 1998 to 2011. For the study, never having used etodolac is defined as a subject never receiving a prescription for etodolac. Active use of etodolac is defined as a subject receiving at least 1 prescription for etodolac within 7 days of the date of their being diagnosed with acute pancreatitis. Non-active use of etodolac is defined as a subject not receiving a prescription for etodolac within 7 days but receiving at least 1 prescription for etodolac ≥ 8 days before the date of their being diagnosed with acute pancreatitis.

Main outcome measure: The association between etodolac use and acute pancreatitis was estimated by using the multivariable unconditional logistic regression model.

Results: After correcting for covariates, the adjusted odds ratio of acute pancreatitis was 3.78 for subjects with active use of etodolac (95% confidence interval 1.11, 12.9), compared with subjects who never used etodolac. The adjusted odds ratio decreased to 1.18 for subjects with non-active use of etodolac (95% confidence interval 0.38, 3.67), but that was without statistical significance.

Conclusion: There could be an association between active use of etodolac and acute pancreatitis. Clinicians should take into account the possibility of etodolac-associated acute pancreatitis when patients currently using etodolac present with acute pancreatitis with an unknown cause.

## 1. Introduction

Etodolac is one of the cyclooxygenase-2 inhibitors, which belong to the nonsteroidal anti-inflammatory agents. Etodolac is available in many countries and is commonly prescribed to treat patients with rheumatoid arthritis, osteoarthritis, and ankylosing spondylitis[1–3]. It also provides relief of pain caused by minor surgery and relief of other types of pain because of its excellent anti-inflammatory and analgesic effects [1, 3]. Etodolac has a favorable safety profile with a low incidence of serious gastrointestinal events, including bleeding, ulceration, and perforation, when compared with other traditional nonsteroidal anti-inflammatory agents [3–6]. However, the U.S. Food and Drug Administration (FDA) has reported that among the 4290 persons taking etodolac who experienced side effects from 2003 to 2012, 27 persons (0.63%) had pancreatitis [7].

Although there is no formal pharmacoepidemiological study available to focus on the association between etodolac use and acute pancreatitis, based on the aforementioned U.S. FDA report, we think there might be an association between etodolac use and acute pancreatitis. If the association really exists, physicians should alert etodolac users to the risk of acute pancreatitis. It is with this in mind that we conducted this case-control study by analyzing the database of Taiwan’s National Health Insurance Program to explore this issue of etodolac use and acute pancreatitis.

## 2. Methods

### 2.1. Study design and data source

Taiwan is an independent country with more than 23 million people. This population-based case-control study was conducted using the database of Taiwan’s National Health Insurance Program. The National Health Insurance Program launched on March 1^st^, 1995, and it covered about 99% of the entire 23 million people living in Taiwan [8]. Further details of the program can be found in previous studies [9–20]. This study was approved by the Ethics Review Board of China Medical University and Hospital in Taiwan. (CMUH-104-REC2-115)

### 2.2. Inclusion criteria

For this study, we selected subjects who were newly diagnosed with acute pancreatitis (International Classification of Diseases 9^th^ Revision-Clinical Modification, ICD-9 code 577.0) during the period of 1998-2011 and aged 20 years or older at the date of their diagnosis. We defined the index date of each case as the date of their being diagnosed with acute pancreatitis. We also randomly selected subjects without acute pancreatitis from the same database as controls who were matched for sex, age (per 5 years), and index year with those subjects who had been diagnosed with acute pancreatitis. We excluded subjects with chronic pancreatitis (ICD-9 code 577.1) or pancreatic cancer (ICD-9 code 157) before the date of their being diagnosed with acute pancreatitis. Comorbidities before the date of subjects being diagnosed with acute pancreatitis that were potentially related to said acute pancreatitis were included as follows: alcohol-related diseases, bililary stone, cardiovascular disease including coronary artery disease, heart failure, cerebrovascular disease, and peripheral atherosclerosis, chronic kidney disease, chronic obstructive pulmonary disease, diabetes mellitus, hepatitis B, hepatitis C, as well as hypertriglyceridemia. All comorbidities were diagnosed with ICD-9 codes. The diagnosis accuracy of the ICD-9 codes has been carefully assessed in previous studies [21–29]. The history of prescriptions for other cyclooxygenase-2 inhibitors available in Taiwan was also collected.

### 2.3. Definition of etodolac exposure

The definition of etodolac use was adapted from previous studies [30–32]. Never having used etodolac is defined as subjects never receiving a prescription for etodolac. Active use of etodolac is defined as subjects receiving at least 1 prescription for etodolac within 7 days of the date of their being diagnosed with acute pancreatitis. Non-active use of etodolac is defined as subjects not receiving a prescription for etodolac within 7 days but receiving at least 1 prescription for etodolac ≥ 8 days before the date of their being diagnosed with acute pancreatitis.

### 2.4. Statistical analysis

We demonstrated the differences in demographic factors, medication use, and comorbidities between the acute pancreatitis cases and the controls by using the *Chi*-square test for categorized variables and the *t*-test for continuous variables. The significant variables found in the univariable unconditional logistic regression model were further included in the multivariable unconditional logistic regression model. The odds ratio and 95% confidence interval were estimated to explore the association of acute pancreatitis with medication use and comorbidities. All data processing and statistical analyses were performed with the SAS software version 9.2 (SAS Institute, Inc., Cary, North Carolina, USA). A two-tailed *P* value < 0.05 was considered statistically significant.

## 3. Results

### 3.1. Characteristics of the study population

Table 1 compares the demographic factors, medication use and comorbidities between the acute pancreatitis subjects and the control subjects. There were 7577 cases of acute pancreatitis and 27032 controls with an even distribution of sex and age. The acute pancreatitis subjects had higher proportions of etodolac use, use of other cyclooxygenase-2 inhibitors, alcohol-related disease, biliary stone, cardiovascular disease, chronic kidney disease, chronic obstructive pulmonary disease, diabetes mellitus, hepatitis B, hepatitis C, and hypertriglyceridemia than the control subjects to statistical significance (*Chi*-square test, *P* < 0.001 for all).

**Table 1:** Chararacteristics of acute pancreatitis cases and control subjects

	Acute ancreatitis				
	Control subjects N = 27032		Cases N = 7577		
Variable	n	(%)	n	(%)	P value*
**Sex**					0.42
Female	9072	33.6	2505	33.1	
Male	17960	66.4	5072	66.9	
**Age group (year)**					0.33
20-39	8149	30.2	2351	31.0	
40-64	13055	48.3	3617	47.7	
65-84	5828	21.6	1609	21.2	
**Age (year), mean (standard deviation)****†**	50.3	15.7	50.1	15.7	0.39
Etodolac					0.004
Never used	27016	99.94	7564	99.82	
Active use	5	0.02	7	0.09	
Non-active use	11	0.04	6	0.08	
Other cyclooxygenase-2 inhibitors					< 0.001
Never used	21490	79.5	5628	74.3	
Have used	5542	20.5	1949	25.7	
Comorbidities before index date					
Alcohol-related disease	119	0.44	339	4.47	< 0.001
Biliary stone	570	2.11	1563	20.6	< 0.001
Cardiovascular disease	4859	18.0	1885	24.9	< 0.001
Chronic kidney disease	481	1.78	322	4.25	< 0.001
Chronic obstructive pulmonary disease	3400	12.6	1176	15.5	< 0.001
Diabetes mellitus	2794	10.3	1509	19.9	< 0.001
Hepatitis B	584	2.16	316	4.17	< 0.001
Hepatitis C	275	1.02	188	2.48	< 0.001
Hypertriglyceridemia	4701	17.4	2252	29.7	< 0.001

Data are presented as the number of subjects in each group, with percentages given in parentheses, or the mean with standard deviation given in parentheses.

* *Chi*-square test and

† *t*-test comparing subjects with and without acute pancreatitis.

### 3.2. Acute pancreatitis associated with etodolac use

After adjustment for covariates, the adjusted odds ratio of acute pancreatitis was 3.78 for subjects with an active use of etodolac (95% confidence interval 1.11, 12.9), compared with subjects who never used etodolac. The adjusted odds ratio decreased to 1.18 for subjects with a non-active use of etodolac (95% confidence interval 0.38, 3.67), but the 95% confidence interval was lacking statistical significance (Table 2).

**Table 2:** Crude and adjusted odds ratio and 95% confidence interval of acute pancreatitis associated with etodolac use.

	**Crude**		**Adjusted** **†**	
Variable	OR	(95%CI)	OR	(95%CI)
Etodolac (never used as a reference)				
Active use	5.00	(1.59, 15.8)	3.78	(1.11, 12.9)
Non-active use	1.95	(0.72, 5.27)	1.18	(0.38, 3.67)

† Adjustment for other cyclooxygenase-2 inhibitors, alcohol-related disease, biliary stone, cardiovascular disease, chronic kidney disease, chronic obstructive pulmonary disease, diabetes mellitus, hepatitis B, hepatitis C, and hypertriglyceridemia

## 4. Discussion

A growing body of epidemiological research has demonstrated that medication use can correlate with the risk of acute pancreatitis [33, 34]. In this present study, we demonstrated that active use of etodolac was associated with a 3.78-fold increased chance of acute pancreatitis, while non-active use of etodolac was not associated with acute pancreatitis (Table 2). These results indicate that only patients who have used and continue to use etodolac might have a risk of acute pancreatitis. Patients who used etodolac before but stopped using it likely do not have the same risk. In further analysis, we demonstrated that acute pancreatitis cases with non-active use of etodolac had longer durations of etodolac therapy than those with active use of etodolac did (mean ± standard deviation, 78 ± 98 *vs.* 14 ± 14 days). This result indicates that the therapy duration of etodolac is not associated with acute pancreatitis, but active use of etodolac is potentially associated with acute pancreatitis. Therefore, physicians should alert patients actively using etodolac about the risk of acute pancreatitis.

There are numerous limitations to this study. The first concern is that drug-induced pancreatitis is an important topic, but there have been no actual reports of etodolac-associated acute pancreatitis worldwide. The U.S. Food and Drug Administration (FDA) reported a casual possible occurrence but did not confirm the causal effect [7]. Likewise, this present observational study cannot fully illustrate the causal effect or the possible pharmacological mechanism connecting etodolac use and acute pancreatitis. Second, there is no evidence to support whether patients who were prescribed etodolac actually took it or not. Thus, our best strategy was to use etodolac prescriptions for analysis. Third, despite active use of etodolac being associated with increased odds of acute pancreatitis, the 95% confidence interval appeared too wide (95% confidence interval 1.11, 12.9). It is our opinion that the number of subjects in our study with active use of etodolac might be too small to narrow the confidence interval (only 7 cases and 5 controls). Also, any major confounders that were not well-controlled would have changed the results of this study. Therefore, further well-designed research with more subjects is needed to confirm our results. Fourth, due to their being no record of indications contributing to etodolac being prescribed and used, there is no knowing whether etodolac was used for controlling acute pancreatitis-related pain. Fifth, a potential bias of this study is that patients using etodolac without a prescription (“over-the-counter”) were missed. Sixth, to present the original differences of comorbidities between the acute pancreatitis subjects and the control subjects, we did not control for said comorbidities. That is why the acute pancreatitis subjects had higher proportions of comorbidities than the controls (Table 1). Seventh, according to the reported data from the U.S. Food and Drug Administration (FDA), the incidence of etodolac-related pancreatitis in the U.S. is quite low, accounting for only 0.63% of patients with etodolac-related adverse effects [7]. It may be that etodolac-related acute pancreatitis is not an interesting clinical issue, yet no pharmacological study is presently available to support this correlation. Indeed, to the best of our knowledge this is the first population-based pharmacoepidemiological study to explore the association between etodolac use and acute pancreatitis in Taiwan. This manuscript is well written and organized and provides updated findings about this issue.

In conclusion, this study demonstrates that there could be an association between active use of etodolac and acute pancreatitis. Clinicians, therefore, should take into account the possibility of etodolac-associated acute pancreatitis when patients currently using etodolac present with acute pancreatitis with an unknown cause.

## Specific author contributions

Kuan-Fu Liao planned and conducted this study. He participated in the data interpretation and also critically revised the article.

Kao-Chi Cheng, and Cheng-Li Lin conducted the data analysis and critically revised the article.

Shih-Wei Lai planned and conducted this study. He substantially contributed to the conception of the article, initiated the draft of the article, and critically revised the article.

## Conflicts of Interest Statement

The authors declare no conflicts of interest.
